# Neoadjuvant degarelix with or without apalutamide followed by radical prostatectomy for intermediate and high-risk prostate cancer: ARNEO, a randomized, double blind, placebo-controlled trial

**DOI:** 10.1186/s12885-018-4275-z

**Published:** 2018-04-02

**Authors:** Lorenzo Tosco, Annouschka Laenen, Thomas Gevaert, Isabelle Salmon, Christine Decaestecker, Elai Davicioni, Christine Buerki, Frank Claessens, Johan Swinnen, Karolien Goffin, Raymond Oyen, Wouter Everaerts, Lisa Moris, Gert De Meerleer, Karin Haustermans, Steven Joniau, P.E.A.R.L. (ProstatE cAncer Research Leuven)

**Affiliations:** 1 Urology, Department of Development and Regeneration, University Hospitals Leuven, Leuven, Belgium; 2 Department of Imaging and Pathology, KU Leuven, Leuven, Belgium; 3 Leuven Biostatistics and Statistical Bioinformatics Center, KU Leuven, Leuven, Belgium; 4 Laboratory of Experimental Urology, Organ Systems, KU Leuven, Leuven, Belgium; 5 Translational Cell and Tissue Research, Department of Imaging and Pathology, KU Leuven, Leuven, Belgium; 6 Department of Pathology, AZ Klina, Brasschaat, Belgium; 7 DIAPath, Center for Microscopy and Molecular Imaging, Université Libre de Bruxelles (ULB), Gosselies, Belgium; 8 Department of Pathology, Erasme University Hospital, Université Libre de Bruxelles, Brussels, Belgium; 9 Laboratories of Image, Signal processing & Acoustics, Université Libre de Bruxelles (ULB), Brussels, Belgium; 10 GenomeDX, Biosciences Inc, Vancouver, BC Canada; 11 KU Leuven, Department of Cellular and Molecular Medicine, Laboratory of Molecular Endocrinology, Leuven, Belgium; 12 Laboratory of Lipid Metabolism and Cancer, Department of Oncology, KU Leuven, Leuven, Belgium; 13 Leuven Cancer Institute, KU Leuven, University of Leuven, Leuven, Belgium; 14 Nuclear Medicine and Molecular Imaging, UZ Leuven, Leuven, Belgium; 15 Department of Radiology Gasthuisberg University Hospitals Leuven, Leuven, Belgium; 16 Department of Radiation Oncology, University Hospitals Leuven, Leuven, Belgium

## Abstract

**Background:**

Recent retrospective data suggest that neoadjuvant androgen deprivation therapy can improve the prognosis of high-risk prostate cancer (PCa) patients. Novel androgen receptor pathway inhibitors are nowadays available for treatment of metastatic PCa and these compounds are promising for early stage disease. Apalutamide is a pure androgen antagonist with a very high affinity with the androgen receptor. The combination of apalutamide with degarelix, an LHRH antagonist, could increase the efficacy compared to degarelix alone.

**Objective:**

The primary objective is to assess the difference in proportions of minimal residual disease at prostatectomy specimen between apalutamide + degarelix vs placebo + degarelix. Various secondary endpoints are assessed: variations of different biomarkers at the tumour level (tissue microarrays to evaluate DNA-PKs, PARP, AR and splice variants, PSMA, etc.), whole transcriptome sequencing, exome sequencing and clinical (PSA and testosterone kinetics, early biochemical recurrence free survival, quality of life, safety, etc.) and radiological endpoints.

**Methods:**

ARNEO is a single centre, phase II, randomized, double blind, placebo-controlled trial. The plan is to include at least 42 patients per each of the two study arms. Patients with intermediate/high-risk PCa and who are amenable for radical prostatectomy with pelvic lymph node dissection can be included. After signing an informed consent, every patient will undergo a pelvic ^68^Ga -PSMA-11 PSMA PET/MR and receive degarelix at standard dosage and start assuming apalutamide/placebo (60 mg 4 tablets/day) for 12 weeks. Within thirty days from the last study medication intake the same imaging will be repeated. Every patient will undergo PSA and testosterone testing the day of randomization, before the first drug intake, and after the last dose. Formalin fixed paraffin embedded tumour samples will be collected and used for transcriptome analysis, exome sequencing and immunohistochemistry.

**Discussion:**

ARNEO will allow us to answer, first, whether the combined treatment can result in an increased proportion of patients with minimal residual disease. Secondly, It will enable the study of the molecular consequences at the level of the tumour. Thirdly, what the consequences are of new generation androgen receptor pathway inhibitors on ^68^Ga -PSMA-11 PET/MR. Finally, various clinical, safety and quality of life data will be collected.

**Trial Registration:**

EUDRaCT number: 2016–002854-19 (authorization date 3rd August 2017).

clinicalTrial.gov: NCT03080116.

## Background

The incidence of prostate cancer (PCa) in the European Union has increased during recent decades since the opportunistic implementation of PSA screening in the clinical practice [1]. Localized PCa is classified in risk groups: low (cT1-T2a, PSA < 10 ng/ml, biopsy Gleason score 6), intermediate (cT2b, PSA10–20 ng/ml, biopsy Gleason score 7), high-risk localized (cT2c, PSA > 20 ng/ml, biopsy Gleason score 8–10) or high-risk locally advanced (cT3–4, cN1) PCa [2]. Fifteen-year cancer-related mortality rate is 20% in intermediate and 36% in high-risk non-metastatic PCa patients treated without curative intent [3]. Conversely, 10-year cancer specific survival for low-risk patients who underwent active monitoring or active treatment is 99% without differences between treatment subgroups [4]. These findings support the notion that lethal disease is rare in the low-risk subgroup. During the last years, the rates of curative treatment for high-risk disease have increased progressively. Conversely, active surveillance has been more and more dedicated to low-risk PCa [5]. However, in the high-risk group, a large part of patients requires other treatments next to radical prostatectomy (adjuvant or salvage radiotherapy, adjuvant systemic treatment) [6]. Considering the increasing application of surgery for high-risk patients, there is an urgent need for studies that assess new treatment combinations in order to maximize cure rates. Treatment of patients with intermediate and high-risk PCa presents two challenges: the need for local control and treatment of possible micro-metastases. Unfortunately, there is still no validated test to detect micro-metastatic disease [7]. Radical prostatectomy with extended pelvic lymph node dissection (ePLND) represents an important therapeutic option within a multimodal approach (adjuvant or salvage radiotherapy, adjuvant systemic treatment) [2, 8].

Neoadjuvant therapy is routinely utilized for the treatment of muscle invasive bladder, esophageal and rectal cancer with the scope of down-staging the primary tumour and control of possible micro-metastatic clones. In this context, neoadjuvant therapy before radical prostatectomy is an interesting possibility in particular for intermediate and high-risk disease. PCa has the peculiarity to be largely dependent on androgen regulation, a mechanism that is routinely targeted in advanced cases. Neoadjuvant hormonal therapy using luteinizing hormone releasing hormone (LHRH) agonists and/or anti-androgens has already demonstrated to downstage primary PCa [9], however, there is a lack of survival data especially for patients with high-risk disease, considering that the previous controlled trials generally assessed low-intermediate risk patients with various ADT regimens and relatively short follow ups [9, 10]. Recently it was suggested that neoadjuvant ADT could decrease cancer related death in high-risk patients, especially when adjuvant EBRT is associated [11]. Considering the increasing availability of new generation androgen receptor pathway inhibitors, there is a new wave of studies that are assessing the anti-tumour effect of these compounds on the primary tumour using pathologic characteristics (pathologic complete response, minimal residual disease) as proxy of an anti-tumour effect [12]. Degarelix is a well-known LHRH antagonist that is commonly used in advanced prostate cancer, with the advantage that it avoids tumour flare-up with a faster depression of testosterone levels compared to LHRH agonists [13]. It was shown that degarelix is not inferior to goserelin + bicalutamide in decreasing the prostate volume in PCa patients [14], thus it was suggested to be a valuable alternative in the neoadjuvant setting. Degarelix was recently studied before prostatectomy but the results were controversial, showing higher intra-tumour levels of dihydrotestosteron compared to LHRH agonist + bicalutamide or degarelix + bicalutamide, generating the hypothesis of unknown mechanisms of action that should be better analyzed in other trials [15]. Interestingly, pathological complete response was obtained for degarelix when it was associated with bicalutamide, generating the hypothesis that a stronger androgen blockade can result in a stronger anti-tumour effect. Degarelix was compared to leuprolide in the pivot trial CS21 in hormone sensitive patients showing a sustained testosterone suppression with improved PSA progression-free survival for degarelix compared to LHRH analogue [16]. In the extension phase, those patients who crossed-over from leuprolide to degarelix, experienced an improved PSA response and progression-free survival supporting the better effect of the LHRH antagonist [17]. Neoadjuvant studies, in the surgical setting, are also a unique opportunity to complete our knowledge on mechanisms of action and molecular response to such new generation of compounds [18]. Apalutamide (alias ARN-509) is a second-generation antiandrogen with a pure antagonist mechanism with 7–10 fold higher affinity with the androgen receptor compared to bicalutamide [19]. It is currently studied in phase III trials for treatment of castration naïve (NCT02489318, NCT02531516) or castration resistant PCa (NCT02257736, NCT02106507, NCT01946204). The possibility to study the anti-tumour effect of the complete androgen blockade based on apalutamide + degarelix is of interest, considering the safety profile of these compounds [16, 20] and potential benefit for the patients.

The therapeutic effect of EBRT is mediated by DNA damage and hence the DNA repair mechanisms are the leading mechanisms in radio-resistance. In preclinical studies, even in the absence of radiation, antiandrogens induce an increased DNA damage and decreased DNA repair in PCa cell lines [21]. Thus, DNA repair is androgen dependent in cell lines, but a better insight in these mechanisms in localized tumour tissue and the assessment of possible novel therapies to decrease the activity of DNA repair mechanisms could help understanding and optimizing treatment modalities. We hypothesize that ARN-509 in combination with degarelix can inhibit DNA repair mechanism more intensively then classic hormonal treatment.

As described above for DNA repair, most studies on androgen-regulated phenomena in prostate cancer are based on preclinical models [22]. We will analyze transcriptomes of pre-treatment biopsies and compare them with the tumor at the moment of radical prostatectomy (after degarelix or degarelix + apalutamide). Transcriptomes will be further complemented with genomic analyses because of known and unknown links between mutations and treatment responses [23].

Second generation androgen receptor pathway inhibitors can induce resistance through different mechanisms [24, 25]. One of these mechanisms is the expression of androgen receptor splice variants. More specifically, the ARV7 variant was found to be a marker for second-generation antiandrogen resistance in mCRPC [26–28]. There is a lack of data about ARV7 expression in primary PCa. Recently it was demonstrated that ARV7 can be expressed in patients treated with degarelix + bicalutamide + abiraterone + prednisolone before prostatectomy but this was not related to the therapy response [29].

PSA is commonly used as a tumour biomarker after RP in order to identify possible recurrences [30]. Early (< 3 year) biochemical failure was identified as a significant predictor of metastatic progression and cancer related death [31], thus representing a useful marker in the clinical practice. We expect an improvement of early biochemical failure in the study arm compared to the control arm. The prognostic value of PSA nadir to predict PCa survival after 3 months of hormonal treatment and before EBRT was tested [32]. PSA levels < 0.5 ng/ml can predict patient survival [32] and this observation was also confirmed recently for PSA levels ≤0.3 ng/ml [33]. PSA nadir before EBRT is, thus, an important biomarker of hormonal response and also a predictor of survival in this setting.

Novel imaging techniques are expected to overcome the problem of invasive sample collection in the near future by allowing accurate diagnosis, tumour staging and assessment of treatment response. Positron emission tomography (PET) is a recognized tool to detect tumour lesions by targeting their metabolic activity or their specific antigen expression. PSMA (prostate specific membrane antigen) is a type II trans-membrane protein that is specifically expressed on the surface of PCa cells and could be used as a tracer to investigate anti-androgen response [34]. PSMA can be targeted by a PSMA-ligand associated to ^68^Gallium. This tracer has been implemented in PET-computed tomography (PET-CT) for the detection of PCa in the metastatic setting [35]. There is also evidence of PSMA regulation by androgens [36]. Immunohistochemical examination of FFPE (formalin fixed paraffin embedded) PCa tissues of patients treated by different anti-androgen therapies showed an over-expression of PSMA after therapy in 55% of primary tumour samples and 100% of metastatic lesions compared to the baseline [37]. Thus, a better insight on PSMA expression in castration naïve patients following new generation ADT is of interest. Recently, PET/MR hybrid imaging has been introduced next to PET/CT in order to allow the combination of specific properties of different imaging techniques.^68^Ga-PSMA PET demonstrated high potential to detect minimal PCa lesions. In preliminary studies, hybrid ^68^Ga-PSMA PET/MR technology has demonstrated to be promising in the diagnosis and follow up of PCa [38]. The same imaging technology might also represent an important improvement for treatment follow-up. The neoadjuvant setting offers an ideal platform to study this hypothesis.

## Methods

### Aim

The primary objective is to assess the residual tumour after surgery. To assess this objective, we aim to evaluate the difference in proportions of patients with minimal residual disease (MRD: tumour volume ≤ 0.25 cm^3^ at final pathology) between the two arms as primary endpoint. MRD and residual cancer burden (RCB) will permit to correct cancer volume by tumour cellularity [39]. The secondary objectives are: a) pathology and immunohistochemistry assessment of the prostatectomy specimen b) whole transcriptome analysis c) exome sequencing d) clinical assessment e) radiological assessment by ^68^Ga -PSMA-11 PET/MR.

### Design and setting

**Fig. 1:**
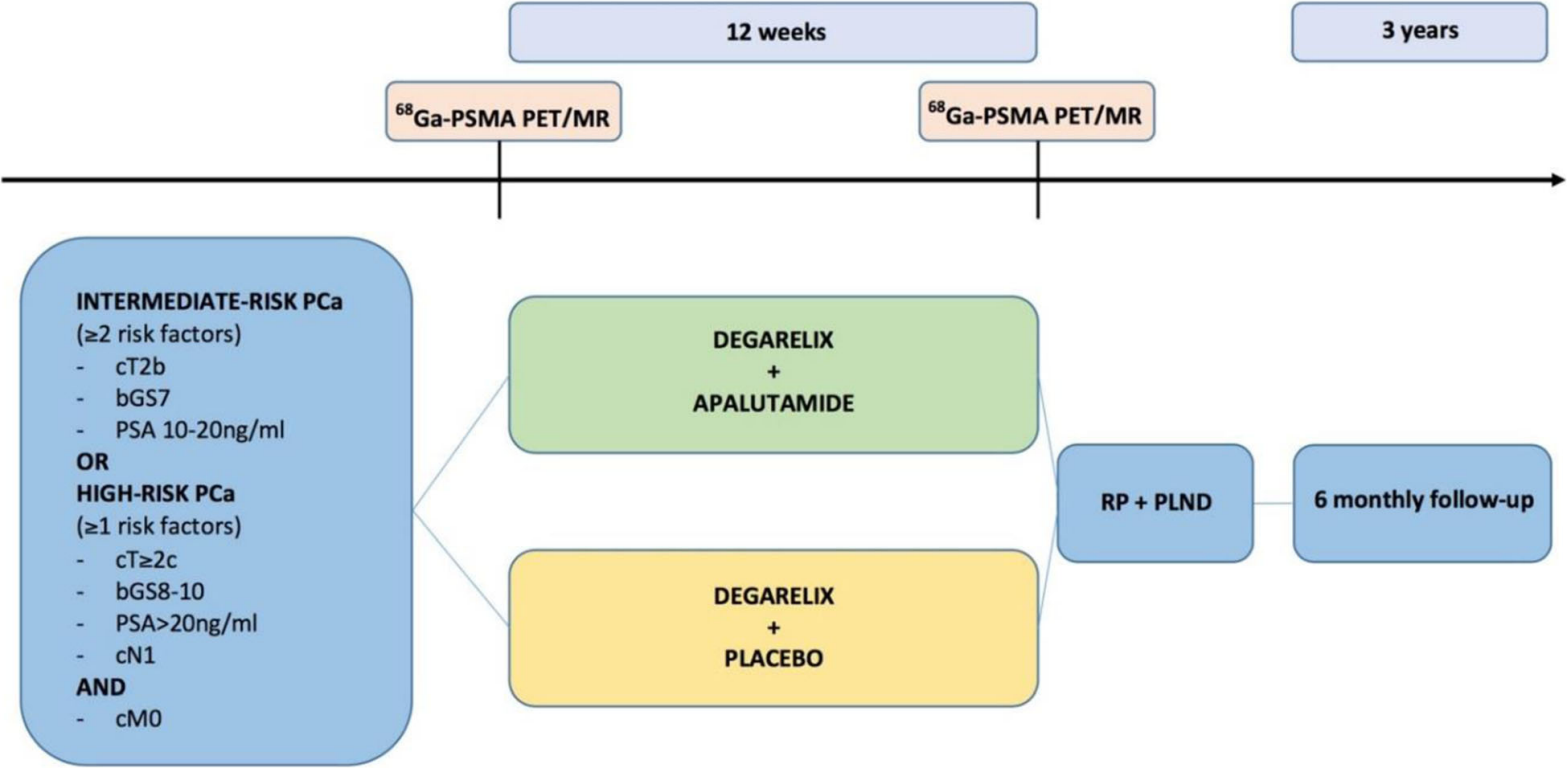
Flow diagram of ARNEO. Ga: Gallium. bGS: biopsy Gleason score. MR: magnetic resonance. PCa: prostate cancer. PET: Positron Emission Tomography. PLND: pelvic lymph node dissection. PSMA: prostate specific membrane antigen. RP: radical prostatectomy

ARNEO is a Phase II, single centre, randomized, double-blind, placebo controlled trial sponsored by UZ Leuven (Fig. 1, Table 1).

**Table 1:** Summary of the main trail activities

Activities	Visits														Perioperative period			
	Screening	V1				V2				V3				V4	T	PO		
Cycles (weeks)		1	2	3	4	5	6	7	8	9	10	11	12					
Randomization		X																
Drugs dispensation		X				X				X								
Informed consent	X																	
Inclusion/exclusion criteria	X	X																
Questionnaires IIEF5, ICIQ EORTC, QLQ-C30		X												X			X	
PSA	X					X				X				X			X	
Testosterone		X												X			X	X
SHBG		X												X				
Blood and urine sampling for research purposes		X												X				
Anestesiological visit														X				
RP + PLND															X			
Charlson comorbidity index		X																
Clavien-Dindo																X		
PSMA PET/MR	X													X				

V: visit. T: treatment. PO: postoperative. SHBG: sex hormone binding globulin

The protocol follows the SPIRIT recommendations for interventional trials [40, 41]. Patients are recruited solely in UZ Leuven from radical prostatectomy candidates who meet the inclusion/exclusion criteria. Patients will be randomized to one of the two arms according to a 1:1 allocation. The ARNEO trial was approved by the Medical Ethics Committee UZ KU Leuven / Research (internal identifier S58827). All participants will provide written informed consent before performing any study-related procedure according to the protocol.

### Participants

Patients with histologically confirmed adenocarcinoma of the prostate without neuroendocrine differentiation or small cell features with 2 intermediate risk factors (cT2b, biopsy GS 7, PSA 10–20 ng/ml) or high-risk (clinical stage≥T2c and/or biopsy GS ≥ 8 and/or PSA > 20 ng/ml and/or cN1) disease can be included. Patients with or without clinical lymph node invasion are both includable but absence of metastatic disease is mandatory. The natural history of non-treated intermediate and high-risk patients suggest that they represent most of the lethal disease [3]. There is increasing evidence that intermediate risk patients, treated with radical prostatectomy, with at least 2 clinical risk factors have poor outcome [42–44]. Thus, these subjects represent an enriched group at higher-risk of cancer specific mortality with similar characteristics as high-risk prostate cancer [42–44].

Patients must have a good performance status (ECOG 0–1), good haematological, renal and hepatic parameters and be amenable for radical prostatectomy + pelvic lymph node dissection by open or robotic approach. Patients are excluded in case of previous surgical/endoscopic treatments of prostatic disease and if assuming food supplements or herbal products that may decrease PSA levels. Exclusion is foreseen in case of other malignancies diagnosed within 5 years before randomization, severe/instable cardiovascular diseases, gastrointestinal disorder affecting absorption and history of seizure or conditions that may pre-dispose to seizure. Patients have to be able to undergo PET/MR imaging.

### Randomization/Stratification/Blinding

The randomization will be centralized and managed by the Leuven Coordinating Centre (LCC). The random sequence is created by a list of random assignments generated by a computer. Allocation of patients to the treatment arms follow a stratified block randomization scheme (with block size of 6). Thus, before the randomization process patients will be stratified according to one of the 2 risk classes: intermediate risk (PSA 10–20 ng/ml, cT2b, bGS7) or high-risk PCa (PSA > 20 ng/ml and/or cT ≥ 2c and/or bGS 8–10 and/or cN1). The randomization will follow an interactive voice response system (IVRS), using a software package to stratify, randomize and allocate patients. The allocation will be managed centrally by LCC that will inform automatically, by institutional email, the pharmacy about the drug assignment. Labelling is done according to the legal requirements to guarantee blinding of participants and personnel. The blinding code can be broken in emergency situations at the discretion of the principal investigator or delegated personnel when knowledge of the study medication has safety implications. Under normal circumstances, the blinding should not be broken until the primary endpoint is reached. Randomization codes will be fully disclosed when the data relative to the primary endpoint are completely collected.

### Interventions

Patients included in the study will be randomly allocated to one of the interventional arms. Before starting the treatment, every patient will undergo a ^68^Ga -PSMA-11 PET/MR of the pelvis (Signa PET/MR system; GE Healthcare, Waukesha, USA using ^68^Ga -PSMA-11 PET/MR). In both arms patients will receive open label degarelix at a dosage of 2 × 120 mg subcutaneously (SC) at first injection, followed by monthly 80 mg SC injections. Subjects will be randomized to receive 240 mg/day of apalutamide (ARN-509; 60 mg 4 tablets/day) or placebo. Within one month from the last oral study medication intake and before the surgical procedure, patients will undergo the second pelvic ^68^Ga -PSMA-11 PET/MR. Treatment period will cover 12 weeks before radical prostatectomy + pelvic lymph node dissection (open or robotic surgery). Patients will undergo the surgical treatment as soon as possible after the last study medication intake, with a maximum delay of 1 month to guarantee the execution of the imaging procedures. Different clinical trials assessed pathologic response to neoadjuvant hormonal therapy comparing treatment length. From the Meta-Analysis of these studies, the longer was the treatment period, the better were the surgical margin and organ confined disease rates [10]. Preclinical data about the effect of apalutamide in animal prostates showed that atrophy, probably driven by apoptosis, was already complete and stable at 28 days of treatment, suggesting an early effect on prostatic tissue [19]. More interestingly, in 28 days, the tumour volume decreased of at least 50% in 75% (6/8) of xenograft mouse models from LNCaP/AR cells and to 100% in 50% of models [19]. However, we considered 3 months as a correct period because this was the most studied and the minimal amount of time in previous comparative neoadjuvant trials with a demonstrated effect.

The lymph node dissection template was previously described [45]: the minimal required template will be an extended (e)PLND. This template includes the external iliac region (boundaries: bifurcation of the common iliac artery proximally, circumflex iliac vein distally, psoas muscle and genitofemoral nerve laterally and medial border of the external iliac vein medially), obturator fossa region (bifurcation of the common iliac artery proximally, pelvic floor distally, obturator muscle laterally, obturator nerve posteriorly, and medial border of the external iliac vein anteriorly) and internal iliac region (bifurcation of the common iliac artery proximally, pelvic floor distally, bladder wall medially, obturator muscle laterally, sciatic nerve posteriorly and obturator nerve anteriorly). The common iliac (boundaries: aortic bifurcation proximally, bifurcation of the common iliac arteries distally, psoas muscle and genitofemoral nerve laterally, and medial border of the common iliac artery medially) and presacral regions (triangle between medial borders of common iliac arteries proximally, line connecting the bifurcations of the common iliac arteries distally, promontory and proximal sacrum (S1–S2) dorsally) are not mandatory but can be included at discretion of the treating surgeon. Postoperatively, patients follow periodic medical examination to ensure safety, collecting clinical data and liquid biopsies (urine, plasma and serum) for future research purposes.

### Measurements

The data collection will be implemented through an electronic case report form (eCRF) that is specifically designed for the study purposes.

1. The primary endpoint is to assess the difference in proportions in MRD between the two arms, correcting for RCB [39]. All pathology evaluations will be done by a dedicated uro-pathologist.
2. Secondary endpoints assessed in this study:
  - Pathological endpoints
    Protein expression in prostatic tumour TMA’s (tissue microarrays) by immunohistochemistry (between treatment arms after radical prostatectomy). Qualitative and semiquantitative evaluation of immunohistochemistry staining will be carried out by a single experienced pathologist. Immunoreactivity will be also quantified using digital image analysis based on two parameters: intensity of immunoreactivity and percentage of immunoreactive cells [46].
    Different biomarkers are assessed: DNA-PKcs and PARP1 (proteins implicated in the DNA repair mechanism), PSA (marker of androgen receptor activity), androgen receptor (tumoural and stromal tissue), Ki67 (marker of proliferative activity), ARV7 (AR splice variant 7 implicated in 2° generation anti-androgen resistance), yH2AX (marker of double strand breaks), PSMA (prostate specific membrane antigen), etc.
    Difference in proportions of pathological downstaging (any decrease in T stage from clinical to pathological stage)
    Complete pathological response rates (no evidence of tumour in the postoperative specimen, pT0)
    Difference in proportions of patients with pN1 disease.
  - Translational endpoints
    Whole transcriptome analysis will follow amplification with optimized reagent chemistry for FFPE extracted RNA and hybridization to 1.4 million feature gene expression microarrays.
    To assess differences at the transcriptome level in pre- vs post-treatment tissues from the same patient
    To evaluate the relationship between transcriptome alterations and clinical outcomes of patients in both arms
    To assess the full exome (exome sequencing)
    To assess copy number aberration profiles between arms
      All transcriptome analysis will be performed by Genome-DX (San Diego, CA, USA).
      Genomic analysis will be performed by the Laboratory of Molecular Endocrinology (KU Leuven).
  - Clinical endpoints
    Differences in:
      ◦ PSA kinetics during and after treatment
      ◦ Difference in proportions of patients with PSA ≤ 0.3 ng/ml after neoadjuvant treatment and before primary treatment.
      ◦ Three-year biochemical recurrence-free survival (biochemical recurrence: PSA > 0.2 ng/ml confirmed by a second PSA evaluation taken at least one week apart).
      ◦ Testosterone changes during the study period
      ◦ Peri-operative parameters (operative time, blood loss, grade of surgical difficulty, etc.)
      ◦ Patient-Reported Sexual, continence and quality of life outcomes based on validated questionnaires (IEEF5, ICIQ EORTC, QLQ-C30)
      ◦ Safety and tolerability will be assessed during the treatment period by collecting adverse events and severe adverse events. Perioperative complications will be assessed by the Clavien-Dindo classification of surgical complications [47].
  - Radiological endpoints
    All radiology evaluations will be done by a dedicated uro-radiologists and specialists in nuclear medicine.
    Maximum and mean Standardized Uptake Value (SUV) in the volume of interest (VOI) defined as index lesion on MR: 1) SUV change (delta) per arm comparing SUV values before and after treatment 2) SUV delta between the two arms.
    Correlation between SUV values and the tumour volume (TV) in the correspondent VOI at definitive pathology.
    Correlation between SUV values and PSMA expression at IHC on the prostate specimen
    Change of MR TV: 1) TV change (delta) per arm comparing TV values before and after treatment 2) TV deltas between the two arms.
    Correlation between MRI TV and TV of the correspondent ROI at definitive pathology.
    Proportion of PI-RADS scores between the arms
    Correlation between PI-RADS score (latest version available) and pathologic Gleason score
    Clinical down-staging and assessment of LNI (proportions of patients with pN1 disease)

### Data analysis

#### Sample size calculation

Sample size calculation was performed to detect 30% difference in proportions of patients in MRD between the two arms with a power of 80% and an α level of 0.05. We consider a difference of at least 30% in proportions of MRD significant because we expect MRD < 20% in the control arm and at least 45% in the treatment arm. This assumption is based on recent data; 20% MRD was observed in patients treated by goserelin + dutasteride [48] probably similar to our control arm. 74% of patients treated with enzalutamide + dutasteride + LHRH analogue [49] had MRD and this group would represent a similar setting as in our study. However, previous data showed 45% MRD in patient treated by abiraterone acetate + LHRH analogue before radical prostatectomy for high-risk PCa [39]. Thus, we set the expected MRD proportion at 45% instead of 74% to be able to detect smaller differences. Calculations are based on a 2-sided Fisher exact test, using SAS PROC POWER software. A sample size of 42 patients per arm is required to verify the main hypothesis. Considering a drop-out of 20%, a total sample size of 102 patients is required. Applying a 1:1 allocation ratio, 51 patients per arm should be included.

### Statistical analysis

The primary endpoint will be analyzed by using an intention to treat (ITT) approach. Per protocol (PP) analysis will be also performed, especially for translational endpoints. For the descriptive statistics, Chi-square test or Fisher exact test will be used to compare proportions and paired t-test (or non-parametric alternatives) to compare pre-post treatment variations. Kaplan-Meier analysis with log-rank test will be used for survival analyses.

The assessment of the transcriptome in tumour tissue pre vs post-treatment and in the two groups will be performed using the mean fold-difference and statistical tests for significance. Unsupervised and supervised clustering analysis will be performed using all the genes or treatment relevant gene networks and pathways. For genome analysis, unsupervised and supervised analysis of expression (*e.g.* hierarchical clustering, heat maps, box plots etc.) will be performed.

## Discussion

ARNEO aims to assess the anti-tumour effect of a complete androgen blockade based on apalutamide + degarelix versus degarelix alone. The rapid decrease of testosterone demonstrated by degarelix obviates the need to add an antiandrogen in the placebo arm which could have masked the effect of the apalutamide, at least at the start of the treatment. The risk for selection bias due to the difference in preoperative tumour volume is compensated by the randomization process and the blinding of participants and personnel to ^68^Ga -PSMA-11 PET/MR. In this study there is the unique possibility to evaluate the molecular response to two ADT regimens at once. From a translational point of view, the possibility to analyze the surgical PCa specimen after neoadjuvant therapy and compare this material to the prostate biopsy, represents a unique opportunity. Preoperative treatment provides a platform to evaluate pharmacodynamics endpoints of novel agents and enable the identification of signals of biologic activity. In addition, it may allow for the identification of molecular and biologic prognostic and predictive factors. In this respect, some promising but mainly preclinical data have been reported on the relationship between androgen receptor signalling and DNA damage response pathways [21, 50–56]. We wish to explore this field through IHC, transcriptome analyses and genome analysis. We chose DNA-PKcs and PARP1 as target proteins for IHC because androgens can activate their transcription [21]. DNA-PKcs is implicated in double strand break (DSB) repair [55]. PARP1 is a nuclear enzyme implicated in DNA repair, tumour proliferation and mediation of androgen receptor-DNA interaction. It can activate the DNA repair process [56, 57]. The therapeutic potential of PARP1 inhibitors has been tested in an explant system of primary human tumours, showing that inhibiting PARP1 enzyme leads to a decrease in tumour proliferation [54]. TOPARP-A (The Trial of PARP Inhibition in Prostate Cancer) phase 2 trial studied the PARP inhibitor olaparib in mCRPC patients showing good response rates particularly for BRCA1/2 or ATM gene-mutated tumours that justified the breakthrough therapy designation by FDA (Food and Drug Administration) [23]. Changes in gene expression after hormonal treatment for PCa have been studied, and have consistently shown a relationship between androgen receptor signalling and DNA repair [50–52]. This link might explain the beneficial effects of androgen deprivation therapy in combination with EBRT, as it is well known that targeting DNA repair mechanisms results in enhanced radio-sensitivity [53]. Although LHRH analogues are commonly used together with EBRT, it is well known that other androgen sources remain active during treatment. Therefore, a certain level of androgen receptor activity remains in PCa cells even under hormonal treatment. This mechanism could sustain the DNA repair mechanisms, potentially causing tumour resistance to ionizing irradiation. The effect of three, 6 and 9 months of neoadjuvant ADT on gene expression in radical prostatectomy samples has been assessed in earlier studies [58]. Medical castration reduced tissue androgens by 75% but also the expression of several androgen-regulated genes including NDRG1, FKBP5, and TMPRSS2; however, androgen receptor and PSA gene expression were not completely suppressed. These data suggest that suboptimal suppression of the androgen receptor axis may be the cause of resistance not only at the primary tumour site but probably also at the level of micro-metastases with important implications in the neoadjuvant treatment of PCa. The use of new generation compounds with more effective androgen receptor pathway inhibition could counteract this remnant androgen activity. The recent LATITUDE phase 3 trial demonstrated that the relative risk of death decreased of 38% in the abiraterone + prednisone + ADT arm vs placebo’s + ADT in metastatic castration sensitive PCa patients [59]. These data suggest that a more effective inhibition of the AR pathway had a therapeutic effect compared to first generation ADT. From this perspective, ATLAS phase 3 trial is randomizing patients to LHRH analogue or LHRH analogue + apalutamide together with EBRT to assess metastasis free survival (NCT02531516).
